# Heparin-Induced Pituitary Apoplexy Presenting as Isolated Unilateral Oculomotor Nerve Palsy: A Case Report and Literature Review

**DOI:** 10.1155/2019/5043925

**Published:** 2019-10-09

**Authors:** Bakr Swaid, Frank Kalaba, Ghassan Bachuwa, Stephen E. Sullivan

**Affiliations:** ^1^Department of Internal Medicine, Hurley Medical Center/Michigan State University, Flint, MI, USA; ^2^Department of Combined Internal Medicine & Pediatrics Residency Program, Hurley Medical Center/Michigan State University, Flint, MI, USA; ^3^Department of Neurosurgery and the Pituitary and Neuroendocrine Center, University of Michigan, Ann Arbor, MI, USA

## Abstract

**Introduction:**

Pituitary apoplexy (PA) is a rare and potentially life-threatening clinical syndrome resulting from pituitary gland hemorrhage and/or infarction. Anticoagulation is a risk factor for triggering PA. Isolated oculomotor nerve palsy is an atypical presentation of PA.

**Case Presentation:**

A 65-year-old African American female with no past medical history of pituitary disease presented to the emergency department (ED) with nonspecific abdominal pain that was thought to be secondary to fecal stasis and subsequently improved with laxatives. She also reported atypical chest pain that was concerning for unstable angina. She was started on aspirin, clopidogrel, and intravenous (IV) heparin. Later, coronary catheterization showed no significant coronary artery disease (CAD). Twelve hours after the procedure, the patient developed acute complete left oculomotor nerve palsy with a severe headache. Magnetic resonance imaging (MRI) of the head showed a large pituitary mass. Pituitary apoplexy was suspected and the patient eventually underwent a successful trans-sphenoidal pituitary resection.

**Discussion:**

We report a case of PA manifesting as isolated left oculomotor nerve palsy without visual field defects in the setting of using dual antiplatelet therapy (DAPT) and IV heparin for acute coronary syndrome. To the best of our knowledge, this unique combination has not been previously reported.

## 1. Introduction

Pituitary apoplexy (PA) is a clinical syndrome resulting from pituitary gland hemorrhage and/or infarction usually in the setting of pituitary macroadenoma. When presenting classically, it is characterized by acute onset headache, vomiting, visual changes, ophthalmoplegia, and loss of consciousness [1]. Asymptomatic pituitary hemorrhage and/or infarction, usually detected by imaging, has been described by some as subclinical PA [2]. However, the term PA is usually reserved for classic PA and thus it remains a clinical diagnosis [1]. Catastrophic hemorrhage into the pituitary gland was first reported in 1898 by Pearce Bailey [3]. The first postmortem description of PA was documented by Bleibtreu in 1905 [3]. The term “pituitary apoplexy” was first coined in 1950 by Brougham to describe the full clinical syndrome [3].

PA complicates 2–7% of pituitary adenomas. When it occurs, it is the presenting symptom/sign in 80% of cases [4]. Most PA cases occur in the context of pituitary adenomas, although rare cases of PA resulting from craniopharyngioma [4], primary pituitary carcinoma [5], and pituitary metastasis [6] have been reported. Most cases of PA occur spontaneously. Sibal et al. published a case series of 45 patients with PA and were able to identify at least one precipitating factor in up to 40% of cases [4]. Hypertension is by far the most common precipitating factor (27%). Other reported triggers include major surgery (especially cardiac surgeries), anticoagulants, head trauma, aspirin, pregnancy, coagulopathies, estrogen therapy, radiation therapy, and dynamic pituitary function tests [3].

The most common ocular complication of PA is visual field deficits (71%) resulting from compression on the optic chiasm [1]. PA is also well-known to result in ophthalmoplegia, in 69% of patients according to a case series reported by Randeva et al. in 1999 [1]. The oculomotor nerve III is the most commonly affected nerve (67%), followed by the abducent nerve VI (29%). The trochlear nerve IV is the least common to be affected (4%), likely as a result of anatomic advantage [1]. The 3rd, 4th, and 6th cranial nerves (CN) all lie in the cavernous sinus and it is common to have two or more nerves affected simultaneously. In fact, cases of bilateral total ophthalmoplegia (third, fourth, and sixth nerve palsies) caused by PA have been reported [7]. In 2007, Lau et al. reported the first case of spontaneous pituitary apoplexy resulting in isolated bilateral oculomotor nerve palsies [8].

Intracranial hemorrhage is a well-known complication of all antithrombotic pharmacotherapy, including antiplatelets (e.g., aspirin), anticoagulants (e.g., warfarin and heparin), and thrombolytics (e.g., streptokinase). Since PA was recognized as a distinct clinical entity, there have been many case reports of PA attributed to the use of classic antithrombotic therapy. More recently, PA resulting from the use of new oral anticoagulants (NOAs) including dabigatran, apixaban, and rivaroxaban [9], have been reported. In this article, we present a case of PA with atypical presentation of isolated third nerve palsy without visual field deficits likely resulting from the use of IV heparin in the setting of a suspected acute coronary syndrome.

## 2. Case Presentation

A 65-year-old African American female with past medical history significant for noninsulin-dependent type 2 diabetes mellitus, essential hypertension, dyslipidemia, chronic back pain, morbid obesity (body mass index (BMI) was 41), gastroesophageal reflux disease (GERD), uterine fibroids, generalized anxiety disorder, and former tobacco abuse presented to the emergency department (ED) complaining of generalized abdominal pain of 3 weeks' duration with intermittent diarrhea and constipation. Upon review of systems, she complained of atypical chest pain which was pleuritic and reproducible on palpation. The chest pain was nonexertional but she had exertional dyspnea. Of note, she had no prior history of cardiovascular disease.

Past surgical history was remarkable for cesarean section and hysterectomy. Prior to admission medications included metformin, hydrochlorothiazide, propranolol, ranitidine, and acetaminophen. Family history was remarkable for diabetes. In terms of social history, she was a former tobacco smoker, drank alcohol socially, and denied illicit drug use. She had a history of allergy to hydroxyzine, propoxyphene, and zomepirac.

In the ED, measurement of vital signs showed a temperature of 37.0°C, heart rate of 88 beats per minute, respiratory rate of 18 breaths per minute, blood pressure of 148/82, and pulse oxygen saturation of 99% on room air. Initial physical examination was normal except for obesity, depressed mood, chest wall tenderness, mild abdominal distention with tympanic note to percussion and mild generalized nonspecific tenderness to palpation. Rectal exam showed small nonbleeding external hemorrhoids. Complete blood count and basic metabolic panel were normal except for blood glucose of 240 milligrams/deciliter (mg/dL). Hemoglobin A1c was 9.3%. Troponin I was slightly elevated at 0.07 and it trended down on second measurement. Electrocardiogram showed normal sinus rhythm with new T-wave inversion in the inferolateral leads. Computed tomography angiography (CTA) of the chest, abdomen, and pelvis was only remarkable for fecal stasis in the rectum. Her abdominal pain and constipation was partially relieved after administration of IV morphine and senna-docusate.

She was also given aspirin for the chest pain. After her abdominal symptoms moderately improved, she was placed in the observation unit for cardiology consultation. After evaluation by the cardiologist, she was admitted to a telemetry unit for an inpatient cardiac stress test. She was receiving 81 mg aspirin daily. On day 2 of hospital admission, she underwent myocardial perfusion scan using the standard 0.4 mg regadenoson (LEXISCAN) injection. The stress test showed reversible perfusion defect involving the apex and lateral wall of the left ventricle, suggesting myocardial ischemia. At that time, clopidogrel (Plavix) was added to aspirin and the patient was started on IV heparin per ACS protocol pending cardiac catheterization. On day 4, coronary angiography was performed and it showed no significant occlusive coronary artery disease (CAD). During the catheterization procedure, the patient received IV heparin and IV nitroglycerin in addition to midazolam for sedation and fentanyl for analgesia. The radio contrast used was iohexol (OMNIPAQUE 350). Post-procedure, IV heparin was discontinued while DAPT with aspirin and Plavix were resumed.

**Figure 1:**
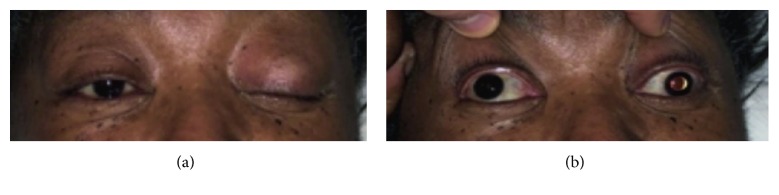
(a) The patient was asked to open both eyes. (b) Both upper eyelids were passively opened. There is a clear anisocoria with down and outward left gaze palsy.

**Figure 2:**
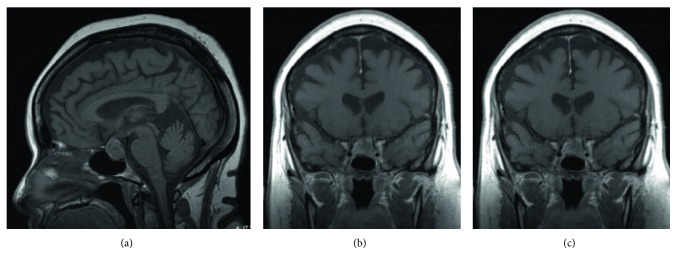
MRI head with contrast T1. (a) Sagittal view showing an enlarged pituitary gland. It measures 1.9 × 2.0 cm in the greatest dimension. The mass is slightly heterogeneous in signal and is slightly impressing on the optic chiasm. (b) and (c) Coronal view with different cuts showing extension on the left cavernous sinus.

After recovery from sedation, the patient complained of a headache which got progressively worse within a few hours. It did not improve with repeated doses of oral hydrocodone and oral/IV acetaminophen. After around 12 hours of cardiac catheterization, the patient developed acute left-sided ptosis, deviated gaze (down and outward palsy), and anisocoria with left pupillary dilation (mydriasis) consistent with acute left 3rd nerve palsy (Figure 1). The patient did not complain of decreased vision and visual field testing by confrontation was normal. She had no other sensory or motor deficits. Her mental status examination did not show evidence of confusion. Her vital signs were normal. STAT CT head without contrast (per stroke protocol) was read as negative for acute intracranial process. CTA of the head and neck was negative for arterial stenosis or aneurysms. The next day, MRI head and orbit with and without contrast were done using Gadodiamide as the MRI contrast agent. The MRI report, as read in our hospital, indicated a nonenhancing heterogeneous pituitary mass measuring 1.9 × 2.0 cm in the greatest dimension and slightly compressing on the optic chiasm suggestive of pituitary macroadenoma (Figure 2(a)). There was no report of any bleed/infarction by either CT or MRI report.

A multidisciplinary team approach was undertaken with involvement of neurology, endocrinology, ophthalmology, otolaryngology (ENT), and neurosurgery. A full ophthalmological examination was normal except for complete left 3rd nerve palsy and mild cataracts. Visual field testing was normal. Hormonal studies were ordered and the results were as shown in Table 1. Of note, prolactin was lower than normal, while adrenocorticotropic hormone (ACTH) level was above the reference range. Both follicle-stimulating hormone (FSH) and luteinizing hormone (LH) were considered low given the patient's postmenopausal status. The patient had normal random cortisol and free T4 level. She did not receive any IV glucocorticoids. The next day after the third nerve palsy, the patient developed a mild fever at 37.9°C with mild neutrophilic leukocytosis (white blood cell count 14.2 K/UL). The patient was transferred to a neuro step-down unit for close observation. Her vital signs remained mostly normal. Subjectively, she neither had a progression of symptoms nor developed new symptoms. Her headache was controlled with oral hydrocodone/acetaminophen. Her full neurologic examination remained normal except for isolated complete left oculomotor palsy. Leukocytosis gradually normalized over several days.

Pituitary apoplexy was highly suspected as a cause of the third nerve palsy, but the diagnosis was challenged by the fact that our radiologist's interpretation of imaging did not mention bleed or cavernous sinus extension. The diagnosis of diabetic neuropathy of the third nerve from microvascular ischemia was contemplated but it was felt less likely because it is usually pupil-sparing [10]. Neurosurgery requested ENT help for trans-sphenoidal pituitary resection. ENT did not feel comfortable with the plan and recommended transfer to a higher level center. The patient and her family were involved in decision making. They were truthfully told that our medical center is not a high volume center for such pituitary cases and the patient might benefit from a transfer to a more experienced center. They chose to transfer and the patient was transferred by ambulance to an academic center of excellence on day 7 of presentation.

**Figure 3:**
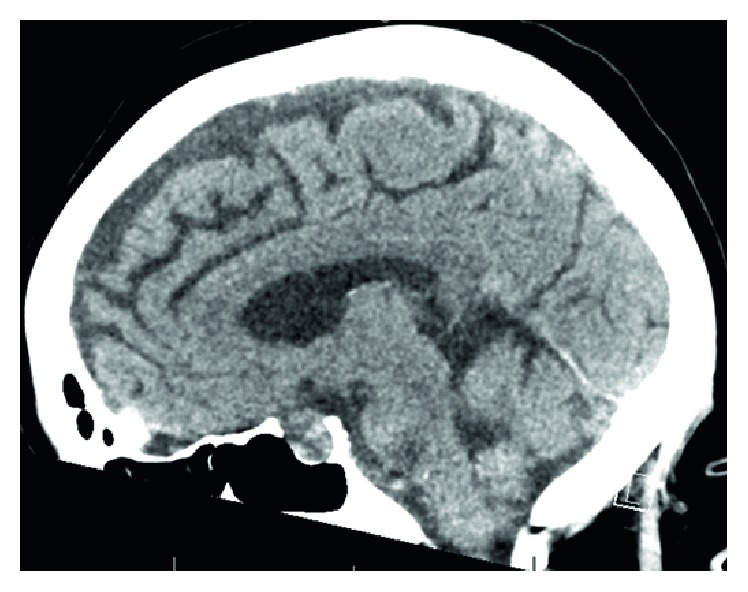
CT head without contrast (sagittal view) showing enlarged sella turcica with increased attenuation suggesting a possible hemorrhage within the tumor.

There, neuroradiologists read the images obtained at our hospital differently. The new CT scan report indicated increased attenuation of the sella turcica and in the suprasellar cistern at the site of the patient's pituitary mass, suggestive of possible hemorrhage within the tumor (Figure 3). The new MRI report indicated that there was a sellar/suprasellar mass lesion which appeared to show central intrinsic T1 shortening and hypoenhancement with peripheral right-sided enhancement. There was mass effect on the optic chiasm superiorly. The lesion appeared to extend laterally into the left cavernous sinus with mild mass effect on the cavernous internal carotid artery. The overall hypoenhancing portion measured 2.1 × 1.9 × 1.3 cm (Figures 2(b) and 2(c)).

The physicians in the other hospital decided to proceed with surgical intervention hoping to relieve the pressure on the third nerve. On day 9 of symptoms, the patient underwent successful trans-sphenoidal pituitary resection. Intraoperatively, the neurosurgeon reported a typical contused apoplectic adenoma. A frozen section biopsy report, as well as final pathology report, confirmed necrotic pituitary tumor with evidence of recent hemorrhage. The apoplectic adenoma was removed and the pituitary gland was left intact. Postoperatively, the laboratory assessment indicated central hypothyroidism and secondary adrenal insufficiency. Vital signs were stable. No major sodium abnormalities were noted. The patient was started on oral hydrocortisone and levothyroxine. Otherwise, the immediate postoperative course was uneventful and the patient was discharged home on day 11 of symptoms development. One-month follow-up at the neurosurgeon's office was remarkable for persistence of oculomotor palsy. A six-month follow-up is scheduled.

## 3. Discussion

In this article, we report a case of a 65-year-old lady with no known history of pituitary disorder prior to admission who suffered from pituitary apoplexy manifesting as severe frontal headache with isolated complete left oculomotor nerve palsy and intact visual fields shortly after coronary angiography, likely resulting from the combination of DAPT and IV heparin given for ACS. To the best of our knowledge, this unique combination has not been previously reported in the literature.

As far as we found, there is no reported case of PA resulting shortly after starting aspirin or other antiplatelets. However, aspirin use was the presumed precipitating factor in 7% of cases of PA according to a case series [4]. The first case of PA resulting from anticoagulant use was reported by Nourizadeh in 1956 [11], six years after Brougham coined the term. It was until 1997, however, when the first case of intravenous (IV) heparin-induced PA was reported by Oo et al. [12]. In 1996, Kelion et al. reported the first case of PA resulting from IV thrombolysis (streptokinase) given for acute MI [13]. In 2007, Tan et al. reported a case of PA triggered by dual antiplatelet therapy (DAPT) along with therapeutic dose of low-molecular weight heparin (LMWH) given for unstable angina in a patient with a known pituitary macroadenoma [14]. The authors raised a concern that treating acute coronary syndrome (ACS) with the combination of DAPT and anticoagulants in the setting of pituitary macroadenoma might be relatively contraindicated.

As mentioned in the previous paragraph, the first case of IV heparin-induced PA was reported in 1997 by Oo et al. [12]. However, that case did not present as CN palsy. Korotinsky et al. reported a similar case to ours in a 68-year-old male with complete right oculomotor palsy following IV heparin and IV nitrates, but their case had an additional clinical presentation with bitemporal superior quadrantanopia which was not present in our case [15]. In 2003, Skljarevski et al. published the first reported case of PA with partial ophthalmoplegia shortly following elective coronary angiography in a patient with atrial septal defect (ASD) in preparation for cardiac surgery [16]. Also in 2003, Nagarajan et al. reported a case of PA with blurry vision, ptosis, and ophthalmoplegia with involvement of the third, fourth, and sixth cranial nerves 36 hours after administering DAPT and therapeutic LMWH for ACS [17]. Moreover, PA stemming from the use of LMWH for thromboprophylaxis in the perioperative period of shoulder arthroplasty was reported by Madhusudhan et al. [18] Again, in that case, the patient had visual deficits and the third nerve palsy was pupil-sparing.

The mechanism of PA is complex and not entirely known [2]. The pituitary gland is a highly vascular organ and pituitary adenomas are known to have increased propensity to bleed when compared to other intracranial tumors [2]. Frequently, pituitary tumors have direct arterial blood supply independent of the hypophyseal portal system that supplies normal pituitary tissue [2]. Also, pituitary adenomas have been shown to have structurally vulnerable blood vessels, decreased angiogenesis, increased sensitivity to glucose deprivation, and high metabolic demand [2]. Hence, PA has been associated with instances of transient hypoperfusion (e.g., major surgeries) or increased metabolic demand (e.g., dynamic hormonal testing and hypoglycemia). The mechanism by which PA results in oculomotor palsy is not entirely known [4]. Suggested mechanisms include direct vascular invasion through the cavernous sinus [8], transmitted pressure from an enlarging sellar mass without extension to the cavernous sinus [4], and interruption of the vasa nervorum supply of the third nerve [19].

In our case, the precipitating factor of PA was likely the use of IV heparin, although many events that happened in our case have been previously reported to trigger PA. These include the use of DAPT [4, 14], iohexol radiocontrast [16], regadenoson [20], IV nitrates use [15], and the coronary angiography procedure itself [16]. A major limitation to our study is the lack of post-operative follow-up and hence the course of pituitary function and third nerve palsy is not known. However, this case has at least three main learning points. First, we should always be cautious when starting IV heparin along with DAPT for ACS, especially if the indication is not very compelling (e.g., atypical chest pain and negative cardiac biomarkers), because of the increased risk of bleeding, including intracranial bleed, and PA. With our patient, unfortunately, IV heparin was commenced 48 hours after ED presentation merely because of a positive stress test despite the patient being free of chest pain at that time. Second, PA can present with a headache followed by isolated unilateral complete third nerve palsy without visual field loss. In contexts similar to our case, a headache could be overlooked because it is a well-recognized side effect of regadenoson and IV nitrates. Therefore, a high index of suspicion is crucial. Third, PA remains a clinical diagnosis supported by radiologic/pathologic evidence. In our case, our radiologist failed to identify bleeding in both the CT and the MRI images. Therefore, when the appropriate clinical context is highly suggestive of PA and the initial radiologist's reading is negative, consultation with a more experienced neuroradiologist is warranted.

## Figures and Tables

**Table 1:** Hormonal workup.

Lab	Result	Reference range
TSH	2.06 *μ*IU/ML	0.3–5.5 micro international unit/milliliter (*μ*IU/ML)
Random plasma cortisol	21 *μ*g/dl	A.M. 4.3–22.4 microgram/deciliter (*μ*g/d) P.M. 3.1–16.7 *μ*g/dL
		P.M. 3.1–16.7 *μ*g/dL
ACTH	50 pg/mL	≤46 picogram/milliliter (pg/mL)
Prolactin	1.1 ng/mL	1.8–20.3 nanograms per milliliter (ng/mL)
FSH	10.4 mIU/mL	Follicular 2.5–10.2 milli-international units per milliliter (mIU/mL), midcycle 3.4–33.4, luteal 1.5–9.1, pregnant <0.3, postmenopausal 23.0–116.3
LH	2.7 mIU/mL	Follicular 1.9–12.5, midcycle 8.7–76.3, luteal 0.5–16.9, pregnant <1.6, postmenopausal 15.9–54.0, prepubertal <6.1, oral contraceptives 0.7–5.6
Insulin-like growth factor (IGF)	157 ng/mL	41–168 ng/mL
Alpha subunits pituitary glycoprotein	0.2 ng/mL	≤1.2 (females premenopausal) ≤1.8 (female postmenopausal)
